# Examining Effectiveness and Predictors of Treatment Response of Pivotal Response Treatment in Autism: An Umbrella Review and a Meta-Analysis

**DOI:** 10.3389/fpsyt.2021.766150

**Published:** 2022-01-27

**Authors:** Mirko Uljarević, Wesley Billingham, Matthew N. Cooper, Patrick Condron, Antonio Y. Hardan

**Affiliations:** ^1^Melbourne School of Psychological Sciences, Faculty of Medicine, Dentistry, and Health Sciences, University of Melbourne, Melbourne, VIC, Australia; ^2^La Trobe University, Bundora, VIC, Australia; ^3^Telethon Kids Institute, University of Western Australia, Perth, WA, Australia; ^4^University Library, The University of Melbourne, Parkville, VIC, Australia; ^5^Department of Psychiatry and Behavioral Sciences, Stanford University, Stanford, CA, United States

**Keywords:** social deficits, language, children, umbrella review, randomized controlled trial, pivotal response treatment, meta-analysis, predictors of outcomes

## Abstract

The current study aimed to provide a comprehensive appraisal of the current evidence on the effectiveness of Pivotal Response Training (PRT) for individuals with autism spectrum disorder (ASD) and to explore predictors of treatment response. We conducted a systematic review of the following electronic databases and registers: PsycINFO, Medline, Embase, Cochrane Central Register of Controlled Trials, ERIC, Linguistics and Language Behavior Abstracts. Six systematic reviews were identified, two with meta-analytic component. Identified reviews varied widely in terms of their aims, outcomes, and designs which precluded a unified and consistent set of conclusions and recommendations. Ten RCTs were identified. Eight of identified RCTs reported at least one language and communication-related outcome. Statistically significant effects of PRT were identified across a majority of identified RCTs for a range of language and communication skills. However, evidence for positive treatment effects of PRT on outcome measures assessing other domains was less robust and/or specific. Overall, both previous systematic reviews and new meta-analysis of the RCTs suggest that PRT shows promise for improving language and communication. Only four RCTs examined the association between baseline child characteristics and treatment outcomes, however, no consistent pattern emerged. This review has identified several key methodological and design improvements that are needed to enable our field to fully capitalize on the potential of RCT designs and characterize detailed profiles of treatment responders. These findings are essential for informing the development of evidence-based guidelines for clinicians on what works for whom and why.

## Introduction

Autism spectrum disorder (ASD) is a cluster of neurodevelopmental disorders characterized by social and communication impairments and the presence of restricted and repetitive patterns of behavior and interests (1). In addition to core symptomatology, a significant portion of individuals with ASD experience a range of additional neuropsychiatric and neurodevelopmental symptoms, cognitive deficits and medical comorbidities (2–6). Although some individuals with ASD have good long-term outcomes (7), a majority continue to experience poor mental health and quality of life with unsatisfactory social, educational and vocational outcomes (8–10). Given the high prevalence, life-long nature and significant public and health costs (11, 12), the development of effective and empirically supported treatment approaches is a crucial priority. Furthermore, where potentially effective interventions are available, a state of the art summaries and critical appraisals of existing evidence is critical for informing and guiding clinical and policy-related decision-making. In addition to establishing an evidence base for the effectiveness of specific treatments, as a necessary step on the path to precision medicine, it is also crucial to understand and characterize profiles of children who stand to benefit the most from a particular treatment, and of children who are unlikely to show significant gains.

Early and intensive interventions based on applied behavior analysis (ABA) and delivered in structured settings have been shown as effective for teaching specific functional skills, reducing problem behaviors, and improving language and intellectual functioning (13–18). However, highly structured ABA approaches may be limited by a lack of generalization of acquired skills (17), high financial cost and time-consuming nature. These concerns have led to the emergence of a group of interventions commonly referred to as the Naturalistic Developmental Behavior Interventions (NDBIs) (19) that combine key ABA principles and techniques with the child-led developmental approach incorporating motivational variables delivered in naturalistic, everyday settings.

Pivotal Response Treatment (PRT) (20, 21) is an NDBI developed to target pivotal areas of motivation, self-initiations, self-management, and responding to multiple cues through the combination of operant learning contingencies, motivational teaching strategies, and child-driven approaches. The rationale behind focusing on noted core developmental areas is that if successfully targeted, they can have a positive effect on a range of other, more specific skills and behaviors (22, 23). Similar to other NDBIs such as the Early Start Denver Model (ESDM) (24), PRT teaching strategies are rooted in the ABA approach and embedded within naturalistic child-adult interactions and designed to enable children to benefit from typical pathways that would not be otherwise available due to the core ASD impairments such as lack of social motivation and attention. One of the key components of the PRT is active parental participation (20) which has been suggested as crucial not only for increasing the number of learning opportunities and overall treatment intensity (25) but also for promoting generalization (26) and beneficial effects on parental well-being (27).

A number of single-subject, small *N* and non-randomized group-based studies have suggested the effectiveness of PRT in ASD (28). For instance, PRT has been shown as effective for improving specific commutation skills such as question-asking, number and length of utterances, speech intelligibility, and spontaneous language, conversation, play, and social initiations (29–32). Furthermore, several studies indicated that PRT led to a reduction in disruptive behaviors (33), anxiety (34) and repetitive behaviors (35).

The lack of randomized controlled trials (RCTs) has been identified as one of the key barriers for progressing the science of ASD behavioral intervention in general (36), and for PRT in particular (37). Therefore, the emergence of PRT RCTs in the last 5 years has been a positive development. It is particularly encouraging that recent RCTs have suggested that PRT outcomes are quite favorable in certain symptom and functional domains, in particular with regards to increase in expressive and receptive language (38, 39) and adaptive communication skills (38). Potentially promising findings for improvements in cognitive functioning (40) and reduction in overall ASD severity (39, 40) also emerged.

Given the increase in the adoption of PRT in clinical practice (21), it is important to systematically appraise existing evidence and achieve a current consensus on the effectiveness of PRT for specific outcomes. It is also crucial to go beyond appraising evidence for group-level effectiveness and provide an in-depth characterization of the baseline characteristics that are associated with positive treatment outcomes. Further, it is important to identify the limitations of the current PRT treatment literature and highlight crucial areas for future improvements. Therefore, we aimed to provide an accessible, state-of-the-art synthesis and integration of current findings on PRT in ASD. The first aim was to conduct an umbrella review of previously published systematic examinations of the literature on the effects of PRT. Although all research designs provide important evidence for the effectiveness of particular treatment practices, RCTs are best equipped for estimating the potential benefits of specific interventions. Crucially, in addition to estimating average treatment effects, if well-powered, RCTs can identify predictors of treatment response and why particular individuals benefit from specific interventions (41, 42). Therefore, the current study aimed to conduct a meta-analysis of PRT RCTs published to date. More specifically, we aimed to (i) investigate the effectiveness of PRT in the domains of core ASD (overall ASD severity, restricted and repetitive behaviors, social and communication abilities) and related (language, cognitive functioning, adaptive behavior, and co-occurring symptoms and behavioral problems) outcomes, and (ii) if enough data were available, to examine predictors of treatment outcomes.

## Methods

Review methodology adhered to the steps described in the Preferred Reporting Items for Systematic reviews and Meta-Analyses (PRISMA) statement (43).

### Information Sources and Search Strategy

Searches were performed in PsycINFO (Ovid) (to May Week 3 2020), Medline (Ovid) (to May 20th, 2020), Embase (Ovid) (to May 20th, 2020), Cochrane Central Register of Controlled Trials (Ovid) (to April 2020), ERIC (Ebsco) and Linguistics and Language Behavior Abstracts (Proquest) by a librarian (PC). All Ovid database searches were conducted on 22nd May 2020 with the ERIC and LLBA searches subsequently run on 25th May 2020. Search terms were developed based on (i) a literature search on ASD and pivotal response treatment and (ii) consultations with the experts in the field and included terms around the broader category of language and behavioral skills training. The broader literature on parental interventions was also examined. No specific subject heading for pivotal response treatment was identified in the included databases, however, the search was made broad by including any mention of the term pivotal. A broad limit was applied to select randomized controlled trials only. The PsycINFO search (Supplementary Table 1) was adapted to the other databases with specific limits and replacement of proximity search operators with Ebsco and Proquest systems. Table 1 shows the key search terms that were used.

**Table 1:** Search terms by domain.

**Category**	**Search terms**
Population	autism, autistic, Asperger^*^, asd, pervasive development^*^, pdd, pddnos
PRT related	Pivotal, prt, naturalistic, communication^*^, development, language^*^, self, self directed, initiat^*^, manag^*^, responsiv^*^, social, behavior^*^, behavior^*^, skill^*^, parent, parents, parental
Treatment	teach^*^, paradigm^*^, intervention^*^, treatment^*^, approach, therap^*^, training, learning
RCT	random^*^, rct, clinical trial^*^, controlled trial^*^, placebo, blind^*^, doubleblind, quasirandom^*^, control group*

* *Abbreviated search term*.

### Eligibility Criteria

Articles published in English were included if they were (a) empirical studies evaluating PRT (manuals and commentaries were excluded but their reference sections were reviewed for relevant empirical papers), (b) published in peer-review journals (conference abstracts and theses were excluded), (c) Randomized controlled trials (RCTs) (other designs including non-randomized studies, controlled before-and-after studies, quasi-experimental and case studies were excluded), and (d) included individuals with ASD (including autism, Asperger's disorder or pervasive developmental disorder not otherwise specified [PDD-NOS] with and without an intellectual disability). No age nor setting (e.g., home, school/kindergarten/other education setting, clinic) limits were imposed. Systematic reviews were excluded from the meta-analysis component but identified for inclusion in the umbrella review component of the study. Reviews without the systematic component were excluded.

### Study Selection and Data Extraction

Following the initial database search, duplicates were removed and study titles were reviewed to remove obviously irrelevant articles. Abstracts of candidate articles were then reviewed for potential inclusion for a full review. Inclusion at this stage only required that the article described a study or review of PRT and ASD. Identified abstracts were removed if they clearly did not meet the inclusion criteria or met one of the exclusion criteria (e.g., single case study, non-randomized trial, etc.). The remaining articles were reviewed in full and evaluated for inclusion/exclusion. The reference sections of the articles were also screened to identify additional articles that might have been missed. Articles that did not meet the inclusion criteria were flagged for removal. Retained PRT RCTs and systematic reviews were coded for the following information:

*Umbrella Review:* (a) type (meta-analysis and qualitative), (b) inclusion/exclusion criteria, (c) period captured, (d) study aims, (e) whether reviews appraised the quality of the included empirical studies, (f) characteristics of included studies (e.g., total *N*; *N* of participants; age), (g) findings regarding outcomes and whether reviews appraised moderators and mediators of treatment outcomes, and (h) specification of limitations of the current research and future directions.

*Empirical Studies:* (a) participant characteristics for both PRT and control groups (e.g., *N*; age; sex, ethnicity, (b) inclusion/exclusion criteria, (c) completion rates, (d) intervention characteristics for both PRT and control group (where applicable) including setting, duration and intensity, (e) dependent variables (primary and secondary), (f) fidelity, (g) outcomes, (h) predictors of treatment outcomes, and (i) country where the study was conducted. Outcomes were narratively summarized and relevant information for the meta-analysis was extracted (for further detail see Analytic Strategy subsection below). Quality indicators for studies and outcomes included in the meta-analysis were assessed based on the assessment protocol utilized by Sandbank et al. (44). Study level indicators included assessment for selection bias (random assignment), blinding and attrition. Measurement level indicators included (i) proximity—whether outcomes were directly taught by the intervention (proximal outcomes) or were developmentally downstream from what was taught by the intervention (distal outcomes), (ii) context—whether assessments were conducted in the same context where the interventions are delivered, for instance, the use of similar materials for both the intervention and the assessment (context-bound) or in the context different from the intervention in terms of setting, assessor or material (generalized), and (iii) the presence of correlated measurement error (CME) which occurs in situations when parents or teachers who deliver interventions also participate in outcome assessments.

### Analytic Strategy

A meta-analysis was performed to consolidate studies examining the effect of PRT on a variety of dependent variables. Where these measures were comparable but not identical, the reported statistics were standardized to facilitate the combination and comparison of the effect estimates. Given that all studies used a baseline/follow-up design and only the mean, standard deviation and sample size were available, a conservative correlation coefficient of 0 (between time points, within pairs) was used—although it is acknowledged that, in practice, this figure is likely to be higher, which would result in increased power and narrower confidence intervals. After a consolidated mean and standard deviation were produced for the treatment and control groups, we used a Student's *T*-Test to determine if there was a significant difference between the PRT treatment group and the control treatment group. A funnel plot and Egger's test of a small study bias were used to examine the evidence of publication bias. Meta-regression was used to investigate the contribution of study-level and quality indications on treatment outcomes.

## Results

### Study Selection

**Figure 1:**
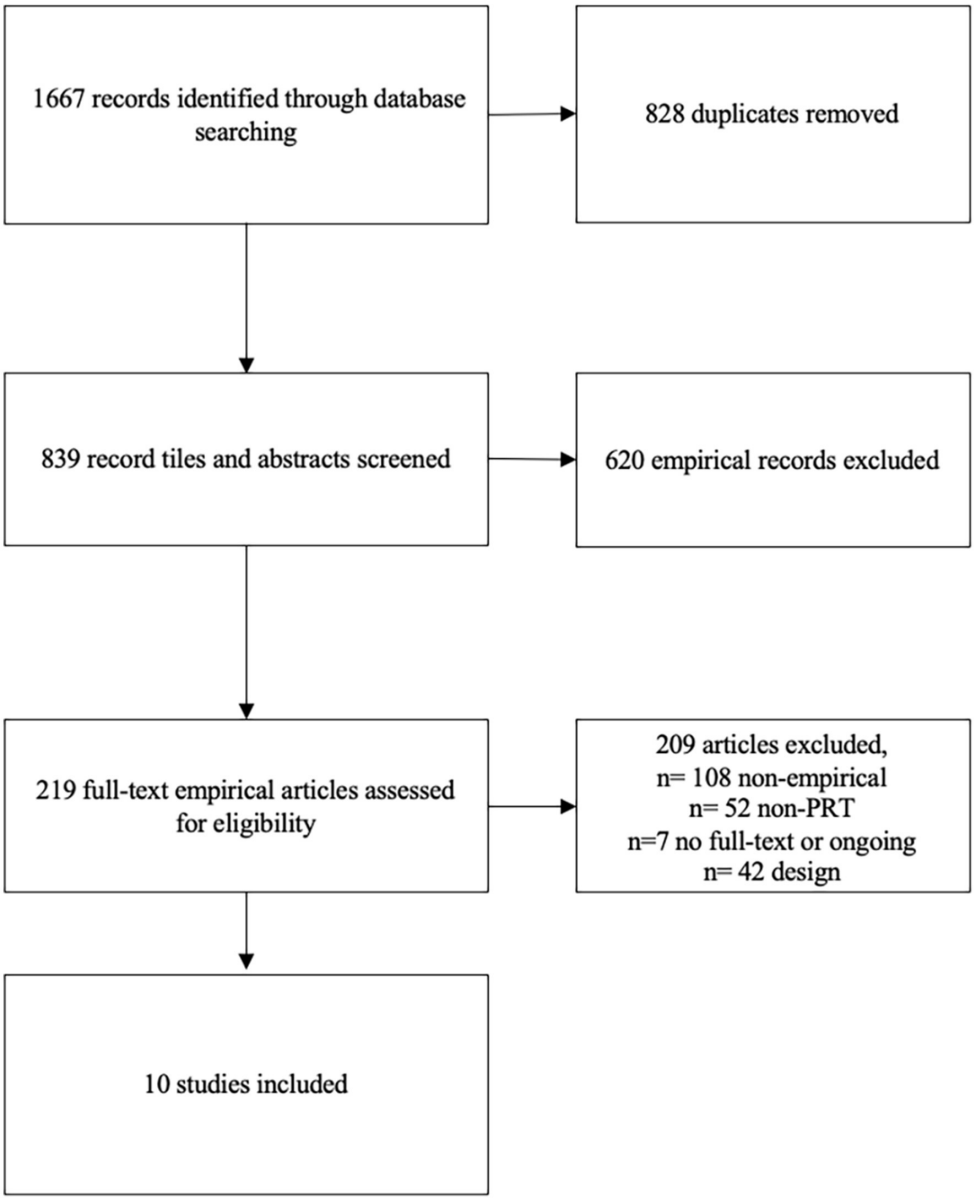
PRISMA flow diagram.

Figure 1 provides an overview of the search results at each stage of the process. Six systematic reviews specifically focusing on the PRT were identified. A Cochrane Systematic Review was also identified (45), however, it was not included as it was still at the protocol stage. Ten RCTs met all the inclusion criteria.

### Umbrella Review

Two reviews included a meta-analytic component (28, 46) and four provided a narrative summary of the studies (37, 47–49). Table 2 provides an overview of the characteristics and findings of the identified reviews.

**Table 2:** Overview of the characteristics and findings from the identified systematic reviews.

**Review**	**Type**	**Inclusion criteria**	**Period**	**Outcomes**	**Aims**	**Study quality**	**Study characteristics**	**Findings summary**
Boudreau et al. (47)	NQ	Peer-Mediated; Age range 4–18 years; Design: no design restrictions	NR	SC	Evaluate peer-mediated PRT for facilitating the SC of school-aged children with ASD	Modified/expanded (by authors) framework for appraising the quality of evidence Reichow et al. (50)	*N =* 5 studies; *N =* 10 participants (8 after removing overlap); Age: 7–10 years; IQ: 55–85	None of the studies met the criteria for classification as promising or established EBP for improving SC impairments
Bozkus-Genc and Yucesoy-Ozkan (28)	M	Design: Single-case; Age range: 1–13 years	1979–2012	No restrictions	Evaluate participant and intervention characteristics, effectiveness and moderators	NA	*N =* 34 studies; Age: 2 years, 5 months-12 years, 8 months; Settings: multiple (44.1%), clinic (26.4%), school (14.7%), home (8.8%), community (5.8%)	Mean PND: 76.10% (SD = 33.65, range: 0–100); effect sizes > 90% in 38.2% of studies, 70–89% in 33.4% of studies, and <70% in 29.4% of studies; PND scores >70% for all of the dependent variables except play and social skills. 14 studies labeled as highly effective, 11 fairly effective, 10 questionable/ineffective. Mean PNCD: 78.03% (SD = 34.38, range: 0-100); effect sizes > 90% in 41.1% of studies, 70–89% in 26.5% of studies, and <70% in 26.4% of studies; PNCD scores >70% for all of the dependent variables except play skills. 14 studies labeled as highly effective, 9 fairly effective, 10 questionable/ineffective. Mean PEM: 89.34% (SD = 22.18, range: 0-100); effect sizes > 90% in 79.4% of studies, 70–89% in 11.7% of studies, and <70% in 8.8% of studies; PEM scores >70% for all of the dependent variables. 27 studies labeled as highly effective, 4 fairly effective, 3 questionable/ineffective.
Cardogan and McCrimmon (48)	NQ	<18 years of age	NR	Study quality	Evaluate adherence of PRT studies to specific research quality standards	Seven specific standards chosen by authors	*N =* 17 studies	Systematic application of an intervention procedure: five studies utilized a pre–post evaluation, 11 multiple baseline procedure, one did not collect any baseline data; Comparison of intervention approaches: two studies compared intervention approaches; Use of standard intervention protocols: 9 studies explicitly aligned with the PRT manuals;
								Treatment fidelity: two studies adhered to the recommended fidelity standard prior to the study start but there were variations during the intervention, five studies no reference to the fidelity measures; Use of objective evaluators: 11 studies used objective evaluators, remaining studies did not reference the use of objective evaluators (two did not require it given the design); Inter-rater reliability: 12 studies reported some form of inter-rater reliability; Longitudinal studies: 8 studies collected follow-up data after the post-treatment stage and 9 did not.
Forbes et al. (49)	NQ	Design: Experimental; Other: at least one communication skill as a dependent variable	1987–2018	Communication	Evaluate primary linguistic and verbal behavior outcomes following PRT and how generalized and collateral outcomes were reported	NA	*N =* 50 studies	The majority of studies aggregated results and/or did not report sufficient detail to determine linguistic forms and/or verbal behavior functions; There was evidence for the generalization of communication skills to untargeted people, settings, materials, and/or activities; Only one study indicated untargeted linguistic forms emerged following PRT and none of the studies described results that indicated improved generalized and collateral verbal behavior functions.
Ona et al. (46)	M	Design: RCT; Age range: ≤ 18 years of age	up to August 2017	SC, SI, RRB	Evaluate social communication, social interaction, and repetitive behavior outcomes in PRT RCTs	GRADE	*N =* 7 studies; *N =* 181 participants; Age: 2.4–9.2 years	Communication (subjective report): two studies, SMD 1.12 (95% CI −0.49; 2.73), *p =* 0.17, GRADE: very low; Expressive language (subjective report): one study, SMD 0.45 (95% CI −0.13; 1.03), *p =* 0.13, GRADE: low; Expressive language (direct measurement): two studies, SMD 0.48 (95% CI.04; 0.93), *p =* 0.03, GRADE: low;
								Receptive language (subjective report): one study, SMD 0.22 (−0.35; 0.79), *p =* 0.45, GRADE: low; Social Interaction: one study (subjective report): SMD 0.48 (−1.10; 1.06), *p =* 0.10, SMD 0.46 (−0.12; 1.04), *p =* 0.12 for CGI-S and SMD 1.12 (0.50; 1.74), *p =* 0.0004 for CGI-I Repetitive Behaviors (direct assessment): one study, SMD 15.97 (95% CI 11.57 to 20.36) p <0.0001, GRADE: low.
Verschuur et al. (37)	NQ	Age: no constraints; Design: no constraints	Up to June 2014	No restrictions	Evaluate: the range of targeted skills; PRT effectiveness for improving children's outcomes; PRT effectiveness for improving parental and staff outcomes and skills; the certainty of evidence; identify limitations and future directions	Quality of evidence (51); Certainty of evidence following classification by Ramdoss et al. (52) into suggestive, preponderant and conclusive	*N =* 37 studies *N =* 420 participants; Age: 1–12.7 years	56.4% of studies had serious methodological limitations; 43.6% of studies showed conclusive or preponderant evidence that PRT increases self-initiations and results in collateral improvements in communication and language, play skills, affect and reductions in maladaptive behavior; The majority of caregivers and staff members were able to implement PRT techniques; Few studies reported on collateral improvements in caregivers' and staff members' behaviors and evidence was qualified as sparse.

*GRADE, Grading of Recommendation, Assessment, Development and Evaluation; M, meta-analysis; NQ, non-quantitative; NR, not reported; PEM, percentage of data points exceeding median; PNCD, percentage of nonoverlapping corrected data; PND, percentage of nonoverlapping data; PRT, Pivotal Response Training; SC, social and communication; SI, social interaction; SMD, standardized mean difference*.

Bozkus-Genc and Yucesoy-Ozkan (28) focused on meta-analytically appraising findings from 34 single case studies to evaluate the effectiveness of PRT across a range of outcomes and identify potential moderators of treatment outcome. Authors found that at least 70% of studies were labeled as highly or fairly effective across dependent variables, irrespective of the method for estimating effect size (e.g., percentage of non-overlapping data [PND], percentage of non-overlapping corrected data [PNCD], and percentage of data points exceeding median [PEM]). Despite positive findings, Bozkus-Genc and Yucesoy-Ozkan (28) also identified a number of methodological limitations. More specifically, treatment integrity, maintenance/generalization, and social validity data were included in only 44, 50, and 25% of studies, respectively.

Ona et al. (46) utilized a meta-analytical approach to evaluate social interaction, communication and repetitive behaviors (RRB) outcomes of 7 studies published before August 2017. The authors were able to synthesize findings for only expressive language and communication outcomes. This analysis supported statistically significant benefits of PRT over control condition for expressive language (2 studies, direct observation; standardized mean difference [SMD]: −0.57, 95% CI 0.04, 0.93, *p* = 0.03), but not for adaptive communication (2 studies, parent and clinician report; SMD: 1.12, 95% CI −0.49, 2.73, *p* = 0.17). At the individual study level, there was evidence for statistically significant benefits of PRT over control condition for RRB (direct assessment) (33) and social interaction clinical global impression-improvement [CGI-I] (38) but not for receptive language (38), communication (subjective report) (53) nor several parental or clinician report measures of expressive language including Vineland Adaptive Behavior Scales (VABS) and MacArthur-Bates Communicative Development Inventories (CDI) (38, 53). The quality of evidence for outcomes was rated as very low for communication and low across other outcomes, based on the Grading of Recommendation, Assessment, Development and Evaluation (GRADE) (54) approach. Several areas for improvement were noted, including the need for a more detailed assessment of implementation fidelity, increased use of validated and objective outcome assessment methods and inclusion of broader outcomes, in particular quality of life and parental stress.

Examining a range of different treatment designs published up to June 2014; Verschuur et al. (37) explored PRT effectiveness for improving children's outcomes, parental and staff outcomes and skills, and evaluated quality and certainty of evidence. Out of 37 identified studies, 35 targeted child behaviors and skills (17 self-initiation, 1 motivation, 31 communication and language skills, 6 play skills, 5 adaptive functioning, 5 maladaptive behaviors, 4 ASD symptom severity, 3 affect, 2 cognitive functioning, 2 academic functioning, 1 face processing, 1 attendance and compliance), 13 targeted parental behaviors (9 implementation fidelity, 2 stress, 2 affect, 2 self-efficacy, 1 empowerment) and 7 staff skills (6 implementation fidelity, 1 effectiveness of training on the ability of staff to conduct assessments). Verschuur et al. found that (i) 43.6% of studies showed conclusive or preponderant evidence that PRT increases self-initiations and results in collateral improvements in communication and language, play skills, affect and reductions in maladaptive behavior, (ii) majority of caregivers and staff members were able to implement PRT techniques, and (iii) collateral improvements in caregivers' and staff members' behaviors were appraised by only a few studies and evidence was qualified as sparse. A number of important areas for improvement was indicated including: (i) need for more experimental and RCT designs, (ii) need for more stringent operationalization and measurement of pivotal skills and collateral outcomes, (iii) characterizing predictors of treatment outcomes and understanding active ingredients of PRT, (iv) understanding parental and staff predictors of effective treatment implementation, and (v) identification of the most effective formats of parental and staff training.

Two systematic reviews appraised evidence of PRT effectiveness for improving communication and/or social skills. Forbes et al. (49) focused on experimental designs by evaluating primary linguistic and verbal behavior outcomes. Boudreau et al. (47) examined peer-mediated PRT for facilitating social-communication behaviors. Interestingly, Forbes et al. (49) noted that the majority of 50 identified studies did not report sufficient detail to enable evaluation of the linguistic forms or verbal behavior functions. Across identified studies, there was evidence for the generalization of communication skills to untargeted people, settings, materials, and/or activities, however, none of the studies described results that indicated improved generalized and collateral verbal behavior function. Using a modified framework for appraising the quality of evidence by Reichow et al. (50), Boudreau et al. (47) concluded that none of the 5 identified studies (10 participants in total across studies) met the criteria for classification as promising or established evidence-based practice for improving social-communication impairments.

Finally, a review by Cardogan and McCrimmon (48) evaluated adherence of 17 identified PRT studies to specific research quality standards selected by authors based on a range of existing quality frameworks. They found that studies showed good quality benchmarks with regards to the use of standardized treatment protocols and application of treatment procedures, inter-rater reliability and objective evaluators. However, variable quality of adherence to treatment fidelity (only 2 studies), comparison of PRT to other approaches (only 2 studies) and collecting follow-up data after the post-treatment stage (8 studies) was observed.

In summary, the reviews undertaken to date covering the period up to 2018 indicate that although PRT can be effective across a range of language and communication outcomes, evidence for other symptom domains and behaviors is limited and that the previous research quality was adversely affected by a range of factors. Importantly, despite the strengths of the previous systematic reviews, they have varied widely in terms of their focus (both with respect to outcome and design) and only two of the reviews included a meta-analytic component and only one focused on RCTs (46). Although the meta-analysis by Nordvik Ona and colleagues was published relatively recently, this review did not capture RCTs published after 2018 and was only able to conduct three meta-analyses, each with only two studies and included one unpublished study (55). Crucially, none of the identified reviews specifically focused on identifying predictors of treatment outcomes. Therefore, it is difficult to form a comprehensive picture of the current state of the literature and the strength of the existing evidence-base for PRT in ASD. Given that four recently published RCTs were not included in any of the summarized systematic reviews, conducting an updated meta-analysis has the potential to provide important additional insights into the effectiveness of PRT.

### Meta-Analytic Review

Ten published RCTs were identified (33, 38–40, 53, 56–60). Importantly, several papers reported data from the same study subjects. Specifically, the two papers by Mohammadzaheri et al. (33, 59) were based on the same sample. The study by Barret et al. (56) reported data from the same subjects as Vernon et al. (40). Finally, the McDaniel et al. (58) paper analyzed the same subjects from the Gengoux et al. (39) RCT. The study by Nefdt et al. (60) was of very low intensity and involved only instructional video material that lasted 1 h and 6 min, therefore, findings were only narratively summarized and were not included in the meta-analysis.

Detailed information on participant and intervention characteristics, dependent variables, outcomes and predictors are provided in Table 3. Table 4 provides a summary of the comparisons between PRT and control groups.

**Table 3:** Overview of the characteristics and findings from the identified randomized controlled trials.

**Study**	**Participants**		**Intervention**		**Dependent Variables**	**Outcomes**	**Predictors**
	**PRT**	**Other**	**PRT**	**Other**			
Barrett et al. (56)	*N =* 12; *M_*age*_* = 35.75 mths, *SD =* 9.31; 8.33% Female; Ethnicity: White (75%), Latino (17%), Asian (8%), Multi-racial (0%).	*N =* 9; *M_*age*_* = 38.22mths, *SD =* 9.78; 11.1% Female; Ethnicity: White (45%), Latino (22%), Asian (11%), Multi-racial (22%).	PRISM Model: Setting: clinician delivered plus parental component; Duration: 6 mths Intensity: up to 10 hrs/w (8 hrs clinician one-on-one; 2 hrs parent education); Mean intensity = 6.81 hrs (25% families met the threshold of 80% completion of all possible treatment hours).	Waitlist	Parent-child play interaction coded for: (i) Parent social bids; (ii) Child social responsiveness; (iii) N total words; (iv) N different words; (v) MLU.	(i) Parent social bids: no significant changes; (ii) Child social responsiveness: significant improvement in PRT (an increase from responsive to 67% of opportunities pre- to 80.9% post-treatment) but not waitlist group; (iii) and (iv) *N* total and different words: not a significant increase in PRT group and no changes in waitlist; (v) MLU: significant increased in PRT but not waitlist group.	The minimally verbal subgroup (*N =* 5) showed large effect sizes (but not statistically significant) for all pre- to post-treatment comparisons. Although at the level of total PRT group initial child responsiveness with caregivers did not show significant association with any of the subsequent outcomes, it was significantly associated with gains in total words, and although no reaching statistical significance, it was moderately associated with gains in different words and mean length of utterance.
de Korte et al. (57)	*N =* 22; *M_*age*_* = 11.87 yrs, *SD =* 1.62; 27.3% Female; Ethnicity: not reported.	*N =* 22; *M_*age*_* = 11.70 yrs, *SD =* 2.11; 31.8% Female; Ethnicity: not reported.	PRT: Setting: seven parent-child sessions, three parent-only sessions, two sessions with involvement of the teacher; Duration: 12 weeks; Intensity: 45 mins per sessions, 90 min per sessions where teachers were involved.	TAU.	Primary: SRS total score; Secondary: CGI; ADOS-2; VABS ABC and subscale scores; Brief Problem Monitor-Parents; Parenting Stress Questionnaire.	(i) SRS total score: significantly higher reduction in PRT vs. TAU on parent-report but not teacher report; (ii) Proportion of responders on CGI-I higher in PRT vs. TAU, however, NS at 12-week and reaching significance at 20-week follow-up (but NS after correction for multiple comparisons); (iii) ADOS-2: NS between PRT vs. TAU; (iv) VABS: NS for VABS ABC, significant improvement in socialization score in PRT vs. TAU; (v) Brief Problem Monitor-Parents: significantly higher reduction on total score in PRT vs. TAU; (vi) Parenting Stress Questionnaire: NS between PRT vs. TAU.	No significant correlations between age, sex and IQ with SRS outcomes; lower symptom severity on ADOS CSS total score associated with higher improvements in the SRS-2 scores in PRT (but not TAU) group.
Gengoux et al. (39)	*N =* 23; *M_*age*_* = 49.5 mths, *SD =* 11.2; 9.5% Female; Ethnicity: White (26%), Latino (17%), Asian (8.7%), Multi-racial (4%), Other (8%).	*N =* 20; Mage= 47.2 mths, SD = 10; 15% Female; Ethnicity: White (30%), Latino (5%), Asian (60%), Multi-racial (0%), Other (5%).	PRT-P: Setting: clinician in home-delivered plus parental component; Intensive phase: Duration: 12 weeks; Intensity: 10h/pw in home clinician delivered; 1h/pw parent training; Maintenance phase: Duration 12 weeks; Intensity: 5h/pw in home clinician delivered; 1h/pm parent training.	DTG	Primary: N functional utterances during 10-min SLO (baseline, week 12 and 24); Secondary: BOSCC; CDI; VABS; PLS-5; MSEL; SRS-2; CGI-S and CGI-I.	Primary: Significantly higher increase in the number of utterances in PRT vs. DTG at both 12 and 24 weeks (primarily driven by the nonverbally prompted utterances); Secondary: Significant treatment effect for BOSCC total and SC scores, CDI (words produced out of 396 and 680), CGI-S, CGI-I (24 months); No treatment effects for PLS-5, MSEL, SRS-2 and VABS.	SLO: age, sex, and baseline characteristics did not predict treatment response; BOSCC: total score: association with lower MSEL scores (predominantly NVIQ).
Hardan et al. (38)	*N =* 25; *M_*age*_* = = 4.1 yrs, *SD =* 1.2; 24% Female; Ethnicity: not reported.	*N =* 23; *M_*age*_* = 4.1 yrs, *SD =* 1.3; 6 Female; Ethnicity: not reported.	PRT-G; Setting: parent delivered; Duration: 12 weeks; Intensity: Eight 90 minute visits (4-6 parents, 1–2 clinicians); Four visits-parent-child dyads with a clinician (60 min).	PEG Duration: 12 weeks; Intensity: Ten 90 minute visits (4-6 parents, graduate student); Two visits-parent-child dyads with a psychologist (60 min).	Primary: N of functional utterances during 10-minute SLO (baseline, week 6 and 12) Secondary: CDI; VABS; CGI-S and CGI-I; SRS; PLS-4.	Primary: In both PRT-G and PEG groups significant improvements in the total number of utterances, improvement higher in PRT-G vs. PEG; Treatment effect most pronounced for imitative and non-verbally prompted utterances, NS for unintelligible and verbally prompted utterances; Fidelity modified treatment effects for total and imitative but not verbally, nonverbally prompted and spontaneous utterances. Secondary: Significant treatment effect for VABS Communication (expressive and receptive) scores, CGI-S and CGI-I scores but not CDI mean length of longest utterance and total words out of 396 and 680, PLS-4 nor SRS total raw score.	Higher age and IQ associated with more total utterances (NS effects for sex); baseline MSEL visual reception a significant predictor of total and imitative utterances. Treatment effect not modified by baseline PLS, CDI nor SRS scores.
McDaniel et al. (58)	*N =* 20; *M_*age*_* = 49.85 mths, *SD =* 11.92; 12% Female; Ethnicity: White (28%), Latino (7%), Asian (56%), Native Hawaiian (2%), Multi-racial/other (7%).	*N =* 20 *M_*age*_* = 46.85 mths, *SD =* 9.66; 12% Female, Ethnicity: White (30%), Latino (5%), Asian (60%), Multi-racial (0%), Other (5%).	PRT-P: Setting: clinician delivered plus parental component; Intensive phase: Duration: 12 weeks; Intensity: 10h/pw in-home clinician delivered; 1h/pw parent training; Maintenance phase: Duration 12 weeks; Intensity: 5h/pw in-home clinician delivered; 1 h/pw parent training.	DTG	Reciprocal vocal contingency derived through an automated process from daylong audio samples from the child's natural environment.	No significant group differences at baseline and 12 weeks but PRT-P had significantly higher-ranked reciprocal vocal contingency scores at 24 weeks (moderate effect size).	NR
Mohammadzaheri et al. (59)	*N =* 15; *M_*age*_* = 110.67 mths, *SD =* 18.71; 40% Female; Ethnicity: Iranian (100%).	*N =* 15; *M_*age*_* = 110.47 mths, *SD =* 18.62; 40% Female; Ethnicity: Iranian (100%)	PRT Setting: clinician delivered Duration: 3 months; Intensity: 60 min per session (child-clinician, parents not present), 2 sessions/pw.	ABA: Setting: clinician delivered Duration: 3 months; Intensity: 60 min per session (child-clinician, parents not present), 2 sessions/pw.	MLU; CCC.	PRT group significantly higher MLU and CCC gains than ABA group	NR
Mohammadzaheri et al. (33)	*N =* 15; *M_*age*_* = 110.67 mths, *SD =* 18.71; 40% Female; Ethnicity: Iranian (100%).	*N =* 15; *M_*age*_* = 110.47 mths, *SD =* 18.62; 40% Female; Ethnicity: Iranian (100%)	PRT Setting: clinician delivered Duration: 3 months; Intensity: 60 min per session (child-clinician, parents not present), 2 sessions/pw.	ABA: Setting: clinician delivered Duration: 3 months; Intensity: 60 min per session (child-clinician, parents not present), 2 sessions/pw.	Disruptive behavior (defined as any behavior that disrupted the session) coded from the videotaped fist and last session (first, middle and last 8 min).	At baseline, PRT group had a significantly higher level of disruptive behaviors; both groups showed a significant decrease in disruptive behaviors with the magnitude of reduction more pronounced in PRT than ABA group (9.9 vs. 1.2 min).	NR
Nefdt et al. (60)	*N =* 13; *M_*age*_* = 38.92 mths, *SD =* 14.57; Ethnicity: not reported in detail, 81% white across both PRT and control group.	*N =* 14; *M_*age*_* = 38.43 mths, *SD =* 11.20.	PRT: Self-directed learning program consisting of education material (DVD lasting 1 h 6 min and manual).	Waitlist	Parental measures: (i) Fidelity of implementation (the following five points were scored: presenting clear opportunities, child choice,	PRT group had significantly higher scores across all dependent variables at posttest that the waitlist group; All parents who completed the self-directed learning program reported high ratings of satisfaction.	NR
					immediate contingent consequences, natural reinforces, reinforcing verbal attempts and correct verbal responses); (ii) Language opportunities; (iii) Observed parental confidence Child measures: Functional verbal utterances		
Schreibman and Stahmer (53)	*N =* 20; *M_*age*_* = 29.5 mths, *SD =* 6.9 10% Female; Ethnicity: not reported.	*N =* 19; *M_*age*_* = 28.9 mths *SD =* 4.2; 15.8% Female; Ethnicity: not reported.	PRT used by parents and therapists to target the development and spontaneous use of functional spoken language. For the first 15 weeks, there were biweekly, 2h parent education sessions (with their child) in the laboratory and additional 2 h/pw child sessions in the home (trained undergraduate student therapists); Additional 8 weeks consisted of five 2 h/pw parent educations sessions and two 2 h/pw in the home with the child.	PECS used by parents and therapists to teach children to use picture icons to communicate; For the first 15 weeks, there were biweekly, 2h parent education sessions (with their child) in the laboratory and additional 2 h/pw child sessions in the home (trained undergraduate student therapists); Additional 8 weeks consisted of five 2 h/pw parent educations sessions and two 2 h/pw in the home with the child.	Spoken Language (MSEL Expressive language scale); Spoken Vocabulary (EOWPVT and CDI); Adaptive Communication (VABS); Parent Satisfaction.	Children in both intervention groups demonstrated increases in spoken language skills, with no significant difference between the two conditions. Seventy-eight percent of all children exited the program with more than 10 functional words; Parents were satisfied with both PRT (rating 5.7 out of 7) and PECS (rating 6 out of 7).	NR
Vernon et al. (40)	*N =* 12; *M_*age*_* = 35.75 mths, *SD =* 9.31 8% Female; Ethnicity: White (75%), Latino (17%), Asian (8%), Multi-racial (0%).	*N =* 11; *M_*age*_* = 34.45 mths, *SD =* 10.08; 18% Female; Ethnicity: White (36%), Latino (27%), Asian (18%), Multi-racial (18%).	PRISM Model: Duration: 6 mths Intensity: up to 10 h/pw (8 h clinician one-on-one; 2 h parent education); Mean intensity= 6.81h (25% families met the threshold of 80% completion of all possible treatment hours).	Waitlist	Primary: ADOS-2; MSEL Composite; PLS-5 Total; PPVT-4; EVT-3; VABS ABC score. Secondary: MSEL (Visual reception, fine motor, expressive and receptive language); PLS-5 (Auditory and expressive comprehension); VABS (Communication, daily living, socialization, motor skills).	For the treatment group, statistically significant changes from baseline were found for all the primary outcomes apart from the EVT-3 and VABS ABC; For the secondary outcomes, there were significant changes for MSEL Visual reception, fine motor and expressive but not receptive language scores, significant changes for VABS communication but not other VABS subscales, no changes for PLS-5 subscales were found. No significant changes from baseline were observed on any measures in the waitlist group for primary outcomes. For secondary outcomes, significant pre-post changes were observed in the Mullen scale of fine motor skills.	NR

*ADOS, Autism Diagnostic Observation Schedule; BOSCC, Brief Observation of Social Communication Change; CDI, MacArthur-Bates Communicative Development Inventories; CGI, Clinical global impression; DTG, Delayed treatment group; EOWPVT, One-Word Picture Vocabulary Test; MLU, Mean length of utterance; MSEL, Mullen Scales of Early Learning; NR, not reported; NS, Not significant; NVIQ, Non-verbal intelligence quotient; PECS, Picture Exchange Communication System; PEG, Psychoeducation group; PLS, Preschool Language Scale; PRISM, Pivotal Response Intervention for Social Motivation; PRT, Pivotal Response Training; SC, social and communication; SI, social interaction; SLO, Structured language observation; SMD, standardized mean difference; SRS, Social Responsiveness Scale; VABS, Vineland Adaptive Behavior Scales*.

**Table 4:** Comparison of treatment effectiveness between pivotal response treatment and control groups.

	**Difference of means**	**SE**	***t***	***p***
SLO	0.39	0.17	2.01	0.09
CDI	0.06	0.21	0.27	0.81
VABS daily living	−0.04	0.25	−0.16	88
VABS expressive	0.41	0.25	1.62	0.26
VABS receptive	0.08	0.84	0.09	0.93
VABS socialization	−0.04	0.28	−0.15	0.89
MSEL expressive	0.03	0.20	0.14	0.89
MSEL receptive	0.05	0.21	0.25	0.81
MSEL composite	0.11	0.25	0.44	0.70
PLS-5 expressive	2.08	2.96	0.70	0.52
SRS-2 total score	−8.09	4.91	−1.64	0.24

*CDI, MacArthur-Bates Communicative Development Inventories; MSEL, Mullen scales of early learning; PLS-5, Preschool Language Scale, Fifth Edition; SLO, Structured Language Observation; VABS: Vineland Adaptive Behavior Scales*.

### Participant Characteristics

Studies included 130 (sample size range 12–25) participants receiving PRT and 122 (sample size range 11–23) children in the control groups. Children's age ranged from 1.5 to 6 years except for Mohammadzaheri et al. (59) and Mohammadzaheri et al. (33) and de Korte et al. (57) who included children older than 6. The percentage of female participants ranged between 8% (40) and 40% (33, 59). No studies provided information on parental and clinician/staff characteristics.

### Intervention Characteristics

Three publications compared PRT to waitlist group (40, 56, 60), two to traditional ABA (33, 59), three to treatment as usual (39, 57, 58), one to parent psychoeducational program (38), and one to the Picture Exchange Communication System (53). Intervention duration varied widely, from one session (60) to 6 months (39, 40, 56). Similarly, intervention intensity varied including 1.5 h in total (60), 2 h of parent education sessions (with their child) in the laboratory, 2 h child sessions in the home per week (53), and a combined weekly parent training session and in-home clinician delivered therapy for 10 h per week for the first 3 months and 5 h during the second 3 months (39).

### Dependent Variables

Eight studies focused on language and communication primary outcomes, utilizing observational coding (38, 39, 56, 59, 60), questionnaire measures such as MacArthur-Bates Communicative Development Inventories (38, 39, 53) and clinician-administered tests such as Peabody Picture Vocabulary Test or Mullen's Scales of Early Learning (39, 40, 53). Only one study (58) utilized an automated coding protocol to assess vocal reciprocity. Four studies assessed social interaction using direct observation (39, 40, 56), clinical global impression scale (38, 39) and parent-report measures such as the Social Responsive Scale (SRS-2) (38, 39, 61). Three studies reported outcomes for adaptive functioning (39, 40, 57). Two studies reported outcomes for cognitive functioning (39, 40) and disruptive behaviors (33, 57), each. Only one study reported effects on parental well-being (57).

### Intervention Outcomes

#### Communication

**Figure 2:**
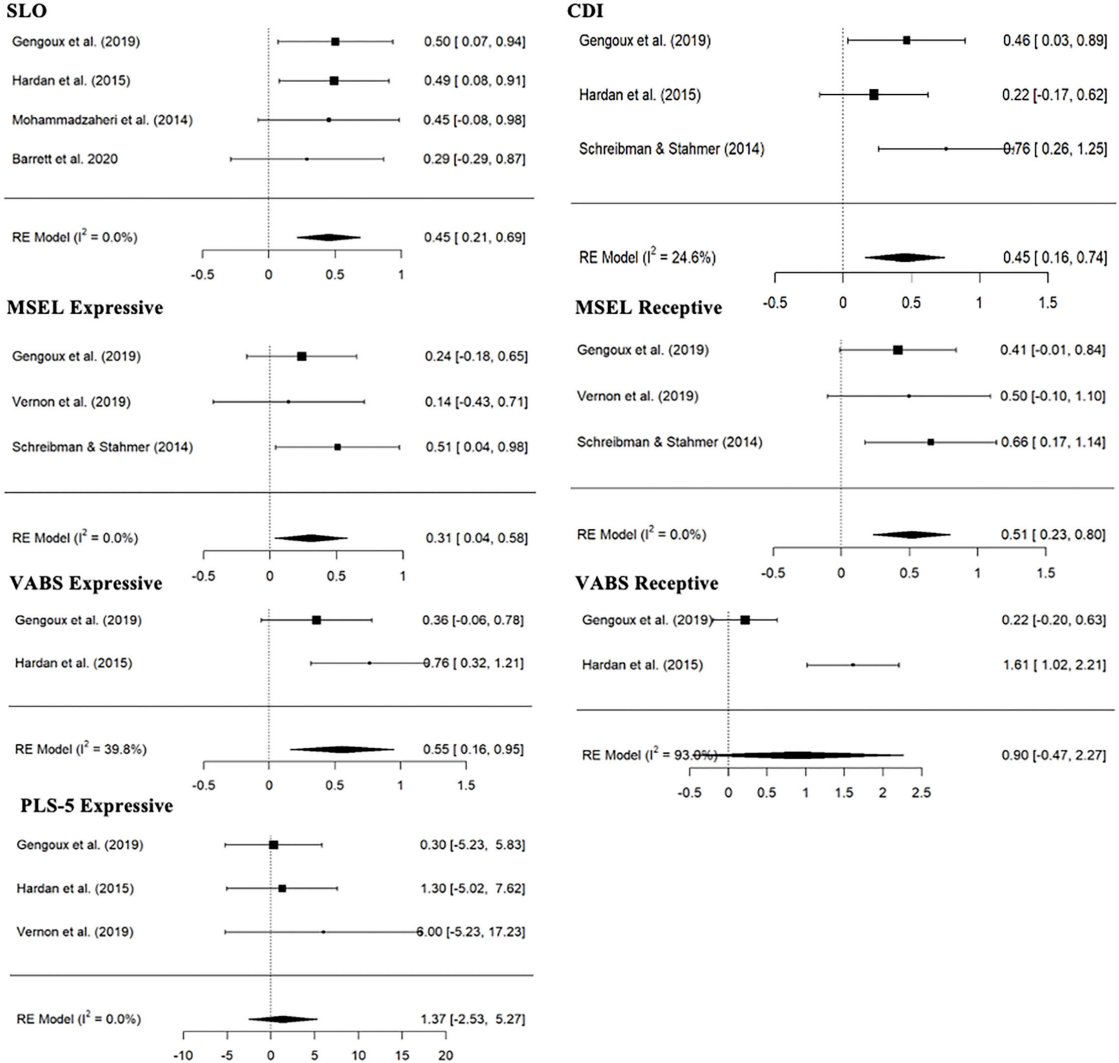
Forest plot of estimates for PRT effects across communication measures. CDI, MacArthur-Bates Communicative Development Inventories; MSEL, Mullen Scales of Early Learning; PLS-5, Preschool Language Scale; SLO, structured laboratory observation; VABS, Vineland Adaptive Behavior Scales.

Figure 2 shows synthesized evidence across a range of communication measures. There was evidence of statistically significant increase from baseline to follow-up in PRT group for structured laboratory observation (SLO) (4 studies; SMD:0.45, 95% CI: 0.21; 0.69), CDI [number out of 680 words CDI score from Gengoux et al. (39) and Hardan et al. (38) and raw number of words from Schreibman and Stahmer (53) were combined] (3 studies; SMD:0.45, 95% CI: 0.16; 0.74), Mullen Scales of Early Learning (MSEL) Expressive (3 studies; SMD:0.31, 95% CI: 0.04; 0.58), MSEL Receptive (3 studies; SMD:0.51, 95% CI: 0.23; 0.80) and VABS Expressive raw score (2 studies; SMD: 0.55, 95% CI: 0.16; 0.95) variables, but not for VABS Receptive raw score (2 studies; SMD:0.90, 95% CI: −0.47; 2.27) and Preschool Language Scale (PLS-5) Expressive score (3 studies; SMD: 1.37, 95% CI: −2.53; 5.27). Wide CI and the heterogeneity prevent strong conclusions with regards to VABS Expressive (*I*^2^= 39.8%), Receptive (*I*^2^= 93.0%) and to a lesser degree CDI (*I*^2^= 24.6%) dependent variables. There were no significant differences between PRT and control treatment groups on any of the treatment outcomes (Table 4). However, the standardized mean change effect estimate for the baseline/follow-up change was higher for the PRT treatment group than the control treatment group (Table 4).

Several studies reported communication-related outcomes that could not be included in the meta-analysis due to non-overlapping measures. Outcome measures ranged from objective assessments such as automated process to derive reciprocal vocal contingency from daylong audio samples from the child's natural environment (58) to parent reports of different aspects of communication such as the Child Communication Checklist and the Preschool Language Scales [e.g., (40, 60)]. Interestingly, positive effects were reported on the majority of these measures (Table 3).

#### Social Interaction

**Figure 3:**
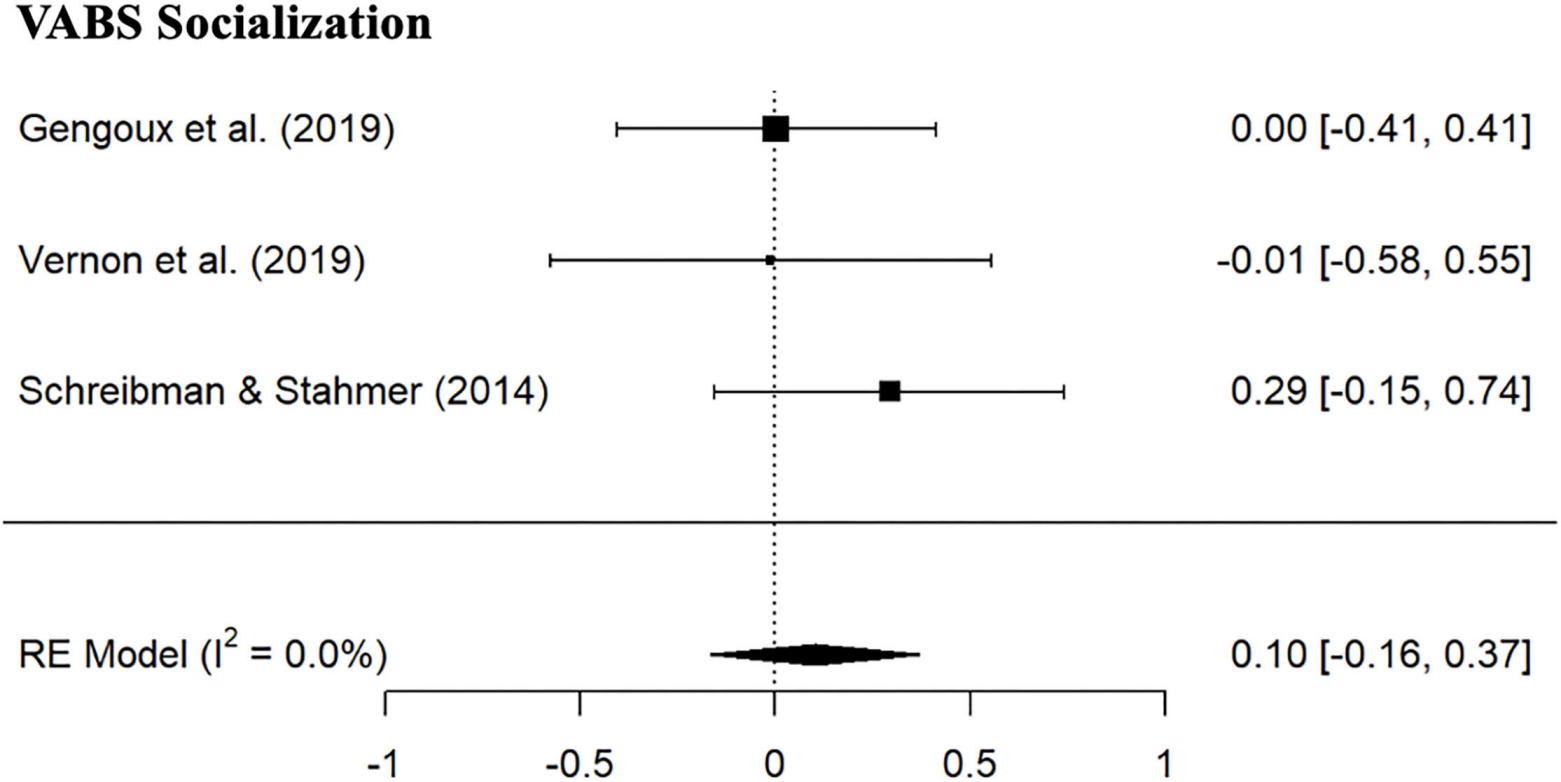
Forest plot of estimates for PRT effects for Vineland Socialization Standard scores.

Given the non-overlapping assessments utilized across the studies, it was possible to combine only VABS Socialization Standard scores for the meta-analysis. As can be seen from Figure 3, there was no evidence of positive effects of PRT (SMD: 0.10, 95% CI: −0.16; 0.37) in the PRT group, and no significant differences between PRT and control group were found (Table 4). Positive treatment effects were reported on CGI improvement social communication subscale (38, 39), the social subscale of the Brief Observation of Social Communication Change (BOSCC) (39) and child social responsiveness coded from parent-child play interaction (56) but not on the SRS-2 social communication raw score (39).

#### Adaptive Functioning and Cognitive Ability

**Figure 4:**
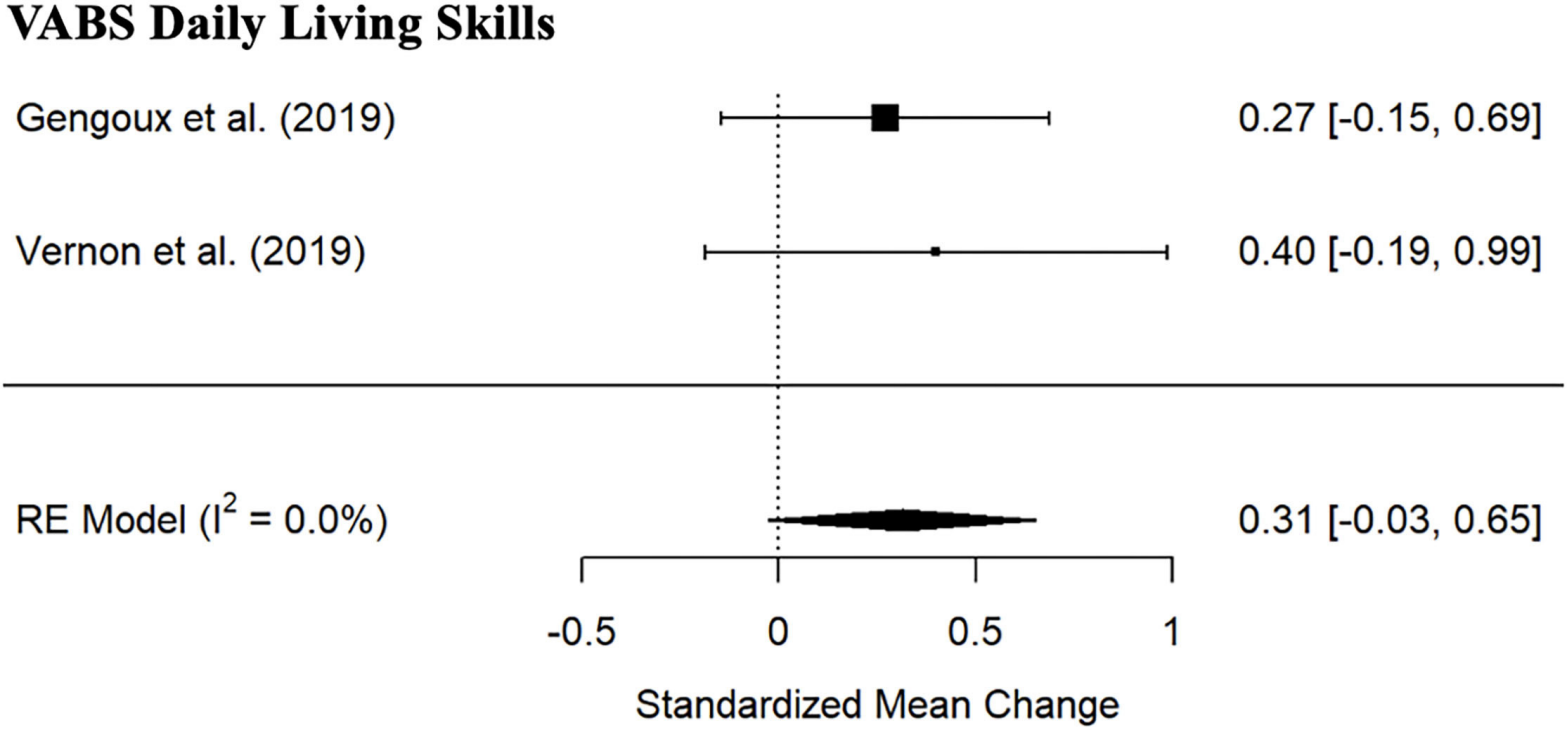
Forest plot of estimates for PRT effects for Vineland Daily Living Standard scores.

**Figure 5:**
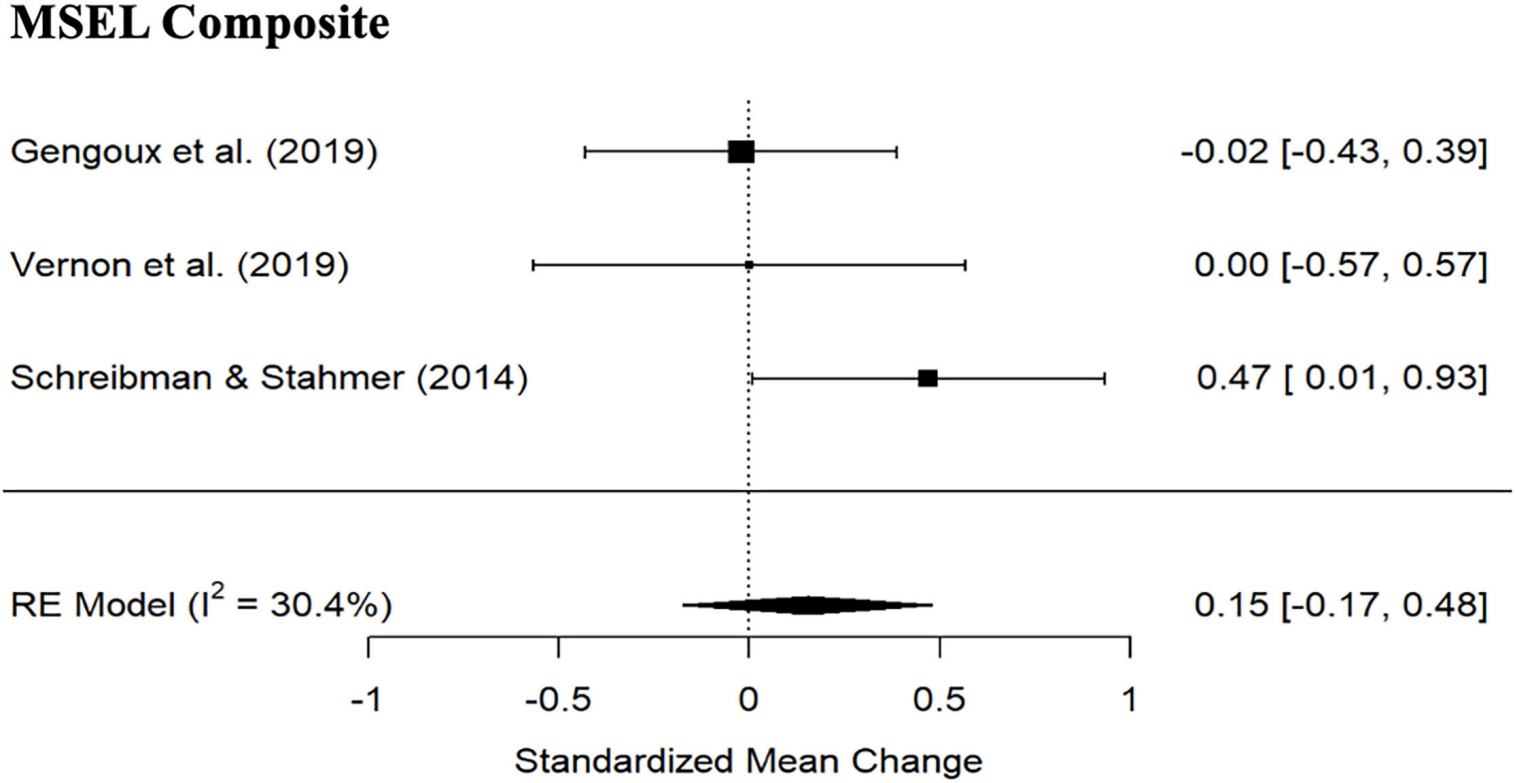
Forest plot of estimates for PRT effects for Mullen Scales of Early Learning composite scores.

Meta-analysis indicated no significant PRT treatment effects for VABS Daily Living skills subscale standard score (Figure 4; 2 studies; SMD: 0.31, 95% CI: −0.03; 0.65) nor MSEL Composite (Figure 5; 3 studies; SMD: 0.15, 95% CI: −0.17; 0.48). Wide CI and the heterogeneity prevent strong conclusions with regards to MSEL Composite (*I*^2^= 30.4%). There were no significant differences between the PRT and the control group (Table 4).

#### ASD Symptomatology

**Figure 6:**
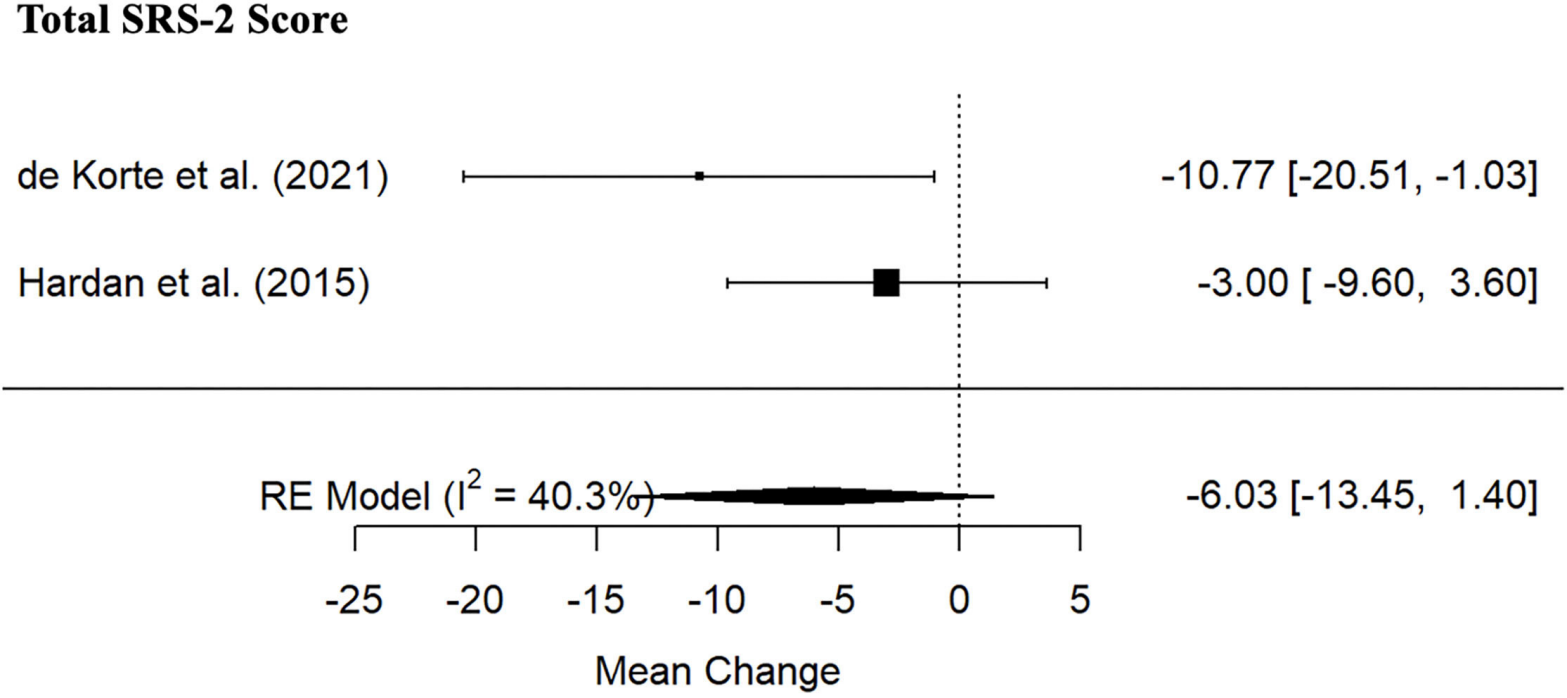
Forest plot of estimates for PRT effects for Social Responsiveness Scale-2 Total Scores.

Meta-analysis indicated no significant PRT treatment effects for SRS-2 Total score (Figure 6; 2 studies; SMD: −6.03, 95% CI: −13.45; 1.40). Wide CI prevents strong conclusions. There were no significant differences between the PRT and the control group (Table 4). Individual studies indicated significant treatment effect on objective indexes of ASD symptom severity such as total BOSCC (39) and the total Calibrated Severity Score of the Autism Diagnostic Observation Schedule (40) (Table 3).

#### Maladaptive Behaviors

Two studies focused on exploring the treatment effects of PRT for the reduction of disruptive behaviors (33, 57). Mohammadzaheri et al. (33) reported that both PRT and ABA groups showed a significant decrease in disruptive behaviors, however, the magnitude of reduction was more pronounced in PRT than ABA group (length of disruptive behaviors reduction was 9.9 min in PRT and 1.2 min in ABA group). Of note, the PRT group had a significantly higher level of disruptive behaviors at baseline. De Korte et al. (57) reported significant reduction in behavioral problems as measured by the Brief Problem Monitor-Parents total score in PRT group.

#### Parental Outcomes

Two studies assessed parental satisfaction with the intervention program. Both Schreibman and Stahmer (53) and Nefdt et al. (60) reported high ratings of satisfaction with PRT (and PECS in the case of Schreibman and Stahmer). Only one study explored effects on parental stress (57) and found no evidence for improvement in this outcome as measure by the Parenting Stress Questionnaire.

#### Treatment Fidelity

All studies reported treatment fidelity with the exception of Mohammadzaheri et al. (33). However, this investigation is based on a similar trial by the same group where fidelity figures were included (59). Across all studies, ≥80% of parents and clinicians reached fidelity at the end of the trial. However, studies did not explicitly report steps taken if interventionists did not meet the standard.

#### Publication Bias

Egger's test revealed no significant (*p* < 0.05) publication bias in any of the language measures. Furthermore, visual inspection of the funnel plots did not indicate any evidence of asymmetry.

#### Outcome Predictors

There was not enough power to conduct the meta-regression. Several individual studies explored predictors of treatment response. Barrett et al. (56) noted that a minimally verbal subgroup (*N* = 5) showed large effect sizes for all pre- to post-treatment comparisons (child social responsiveness, number of total and different words), but the difference did not reach significance when compared to the verbal subgroup. In addition, within the minimally verbal group, initial rates of child responsiveness were strongly associated with subsequent gains in total words and moderately, but not significantly, associated with gains in different words and mean length of utterance; no significant associations were identified at the whole group level. Gengoux et al. (39) found that age, sex, and other baseline characteristics including developmental level (MSEL score) did not predict changes in treatment effect on any of the outcomes, however, the lower MSEL score, in particular the non-verbal subscales, were significantly associated with greater improvement on the BOSCC total score. Hardan et al. (38) found that while higher baseline MSEL visual reception scores were a significant predictor of treatment response for total and imitative utterances, the treatment effect was not modified by sex nor baseline PLS, CDI nor SRS scores. De Korte et al. (57) found no significant correlations between age, sex and IQ with SRS outcomes, however, they reported that lower symptom severity on ADOS CSS total score associated with higher improvements in the SRS-2 scores in PRT (but not TAU) group.

#### Quality Indicators

Study level quality indicators are presented in Supplementary Table 2. There was incomplete/insufficient information to ascertain (i) randomization in three studies (33, 59, 60), (ii) blinding in two studies (40, 56), (iii) attrition in one study (60). Effect size-specific measurement quality indicators are presented in Supplementary Table 3. Majority of studies utilized distal measures and generalized contexts with low correlated measurement error bias.

## Discussion

The current study aimed to provide a comprehensive appraisal of the current evidence on the effectiveness of Pivotal Response Treatment (PRT) for individuals with ASD through an umbrella review of previous systematic examination of the literature and meta-analytic synthesis of all available randomized controlled trials (RCT) of PRT. Overall, both previous systematic work and a new meta-analysis of the RCTs suggest that PRT shows promise for improving language and communication. However, evidence for improvements in other areas is less strong. Crucially, only three studies examined predictors of intervention outcomes.

Our umbrella review captured six systematic examinations of the literature specifically focusing on PRT, two with a meta-analytic component and four providing a descriptive summary of the findings. These reviews varied widely in terms of their aims, outcome, and designs. One of these studies aimed at appraising treatment effectiveness (37) while another focused on adherence to specific research quality standards (48). One review aimed to capture comprehensive outcomes across a number of domains (37) while another one targeted communication only (49). Reviews captured different designs with one focusing on single case reports (28), one on RCTs (46), and another on a combination (37). Therefore, it is difficult to form a unified and consistent set of conclusions and recommendations. However, several observations can be made. The majority of the reviews encompassing all study designs provided evidence that PRT was effective for certain aspects of language and communication (28, 37, 46, 49). Importantly, the positive effects of PRT were observed across assessment methods. The only exception was a systematic review by Boudreau et al. (47) that concluded that, based on the criteria put forward by Reichow et al. (50), none of the included studies could be classified as promising or established for improving social-communication impairments. However, Boudreau et al. (47) included 5 single case studies (with 10 participants in total) that focused only on peer-mediated PRT which may explain the lack of significant treatment effects. Other outcomes (e.g., adaptive functioning, cognitive functioning, overall ASD severity) were less frequently appraised and therefore it is difficult to ascertain evidence of PRT effectiveness for non-language/communication outcomes. Additionally, each review raised a range of limitations of the identified studies that can be systematized into the following three broad categories. The first is related to the nature and comprehensiveness of appraised outcomes and the type of assessments, in particular the need to incorporate more objective measures and capture parental outcomes. The second category included the lack of understanding of predictors of response and active treatment ingredients. Finally, the third group is related to the lack of understanding of parental and staff predictors of effective treatment implementation.

Eight of ten identified RCTs reported at least one language and communication-related outcome and it was possible to conduct six meta-analyses across different measures with a number of synthesized studies varying between two (for VABS expressive and receptive subscale), three (for CDI, MSEL expressive and receptive subscale, and PLS-5 expressive subscale) and four (SLO for assessment of utterances). Our meta-analysis indicated clear benefits in language abilities from PRT. A statistically significant increase from baseline to follow-up in the PRT group was observed for both objective (SLO, MSEL expressive and receptive scores) and parent- and/or clinician-report (CDI, VABS expressive score) measures of language and communication. However, no differences from baseline were observed on the VABS receptive scale and the PLS-5 expressive scale. A range of other language and communication outcomes that could not be synthesized in the meta-analysis also indicated positive treatment effects. These encompassed positive effects on both the parent- and/or clinician-reports including the Children's Communication Checklist (CCC) (59), the One-Word Picture Vocabulary Test (EOWPVT) (53), the Peabody Picture Vocabulary Test (PPVT-4) (40) and automatic coding of vocal reciprocity (58). The only exception was the Expressive Vocabulary Test (EVT-2) total score that did not significantly improve as a result of PRT (40).

PRT studies have also examined a range of non-language target behaviors. Five studies to date used outcome measures to assess overall ASD severity, adaptive functioning, cognitive functioning, and disruptive behaviors. Only one meta-analysis, although limited by the number of studies, was possible for social interaction (2 studies, VABS Socialization scale), overall autism symptom severity (2 studies, SRS-2 Total Score), adaptive functioning (2 studies, VABS Daily Living scale) and cognitive functioning (3 studies, MSEL Composite) each, indicating no significant PRT treatment effects for these outcomes. A wide CI and considerable heterogeneity prevent strong conclusions regarding cognitive functioning. It is also important to highlight that results from individual studies that were not possible to be synthesized in meta-analysis suggested significant treatment-related improvements for social interaction measured by CGI (38, 39), BOSCC social subscale (39) and parent-child interaction coded for social responsiveness (58), but no effect on the SRS-2 Social Communication and Interaction raw score (39). Similar improvements related to overall ASD symptoms were observed as measured by the BOSCC total score (39) and Autism Diagnostic Observation Schedule (ADOS) Calibrated Severity Score (CSS) total score (40), however, meta-analysis based on two studies (38, 57) indicated no significant positive effects for the SRS-2 total score (38). Finally, positive treatment effects of PRT were also reported in reducing the magnitude of disruptive behaviors (33, 57).

Only four studies examined predictors of treatment outcomes. Although not significant, the findings reported by Barrett et al. (56) provide some evidence that a minimally verbal subgroup (*N* = 5) might show better treatment response compared to the verbal subgroup. Further, Barrett et al. reported that, while initial rates of child responsiveness were not predictive of subsequent outcomes at the group level, they were associated with subsequent gains of vocabulary in the minimally verbal group. Hardan et al. (38), De Korte et al. (57), Gengoux et al. (39) found that age and sex were not related to subsequent outcomes; however, they reported inconsistent findings with regards to the effects of IQ. More specifically, while Gengoux et al. (39) found that the lower MSEL score, in particular the non-verbal scores, were significantly associated with greater improvement on the BOSCC total score, Hardan et al. (38) found that higher baseline MSEL visual reception scores were a significant predictor of treatment response for total and imitative utterances and de Korte et al. (57) reported no significant associations between IQ and outcomes. De Korte et al. (57) reported that lower severity of autism symptoms at the baseline was associated with higher improvements in the SRS-2 scores in PRT group. Therefore, based on the currently existing evidence, it is not possible to identify a consistent pattern of baseline characteristics that are associated with PRT treatment outcomes.

### Limitations and Future Directions

Despite a notable increase in the number of PRT RCTs in the last few years, identified studies were all limited by small to moderate sample size, a significant limitation that needs to be taken into account when appraising the current body of evidence for the effectiveness of PRT. When interpreting the comparisons of the effectiveness between PRT and control groups, it is important to note that due to the limited number of RCTs identified, it was not possible to conduct separate analyses for RCTs that used active (e.g., ABA) and waitlist control groups. In addition, this systematic review has identified several other key limitations that should be addressed in future research. Firstly, future studies will need to include more comprehensive treatment targets, in particular adaptive functioning, a generalization of treatment effects and longer-term (12-months or longer) outcomes. In addition, only one of the identified RCTs have explored the effects of PRT on parental well-being, reporting no significant beneficial effects on parental stress (57). Comprehensive understanding of the effects of PRT on parents, both direct and indirect, is particularly crucial given that high levels of stress, anxiety and depression and poorer quality of life among parents of children with ASD are well established (62–66). It is encouraging that several RCTs have shown positive treatment effects on objective measures, therefore reducing the risk of bias, however, this approach should become a standard practice for future studies. Additionally, it is well recognized that a range of currently available, standardized ASD diagnostic and quantitative severity measures such as the Autism Diagnostic Interview-Revised (ADI-R), the ADOS and the SRS-2, have limited sensitivity to change and response to interventions (67) which restricts their utility in the context of clinical trials. Recently, the Brief Observation of Social Communication Change (BOSCC) has been developed as a measure for capturing the change of core ASD symptoms. Despite promising initial findings (68–70), the BOSCC RRB domain appears to be less sensitive to changes (39). Therefore, further development of instruments able to capture the subtle change in distinct symptom domains is an area of urgent need.

Individual differences in treatment response among individuals with ASD are well established (71–73). Although baseline characteristics such as gross measures of cognitive and language level and overall ASD severity have been found to predict response across a range of existing treatments (74, 75), our field lacks a comprehensive understanding of specific factors underlying individual variability in response to particular intervention and treatment components and is therefore missing crucial information for enabling individualization of treatments (76, 77). One of the major benefits of well-powered RCTs is the ability to characterize predictors of treatment response and how and why specific interventions benefit individuals with ASD (41). However, although four identified PRT RCTs have explored predictors of treatment outcomes (38, 39, 56, 57), a combination of sample size, analytic and methodological limitations did not allow us to conduct meta-regression and gain more robust insights into specific predictors of PRT response. Therefore, it will be crucial for future PRT RCTs to improve trial methodology by adopting factorial designs, comparative efficacy trials and adaptive treatment designs while implementing more advanced individual difference analytical strategies that would enable the identification of subgroups of children who respond well to PRT and understand the profile of treatment responders.

## Conclusions

Statistically significant effects of PRT on a range of language and communication skills were identified across a majority of ten RCTs included in this review. This finding is in line with the hypothesis that increasing social motivation and thus the quality and quantity of opportunities for social learning will yield positive downstream effects on language and communication abilities (21). However, evidence for positive treatment effects of PRT on outcome measures assessing other domains was less robust and specific. This review has identified that several key methodological and design improvements are needed to enable our field to fully leverage the potential of RCT designs and establish not only overall treatment efficacy but, more importantly, detailed profiles of treatment responders and therefore provide evidence-based guidance for clinicians on what works for whom and why.

## Data Availability Statement

The original contributions presented in the study are included in the article/Supplementary Material, further inquiries can be directed to the corresponding author/s.

## Author Contributions

MU and AH designed the study. PC and MU conducted the systematic search. MU and AH screened the eligible studies and extracted the data. WB, MC, and MU conducted the analyses. MU, AH, WB, and PC drafted the initial manuscript. All authors critically reviewed, provided feedback on the initial version of the manuscript, and approved the final version of the manuscript.

## Funding

This work was supported by National Institute of Mental Health (R21DC01608902) to AH. MU is currently supported by the Australian Research Council Discovery Early Career Researcher Award (DE180100632).

## Conflict of Interest

The authors declare that the research was conducted in the absence of any commercial or financial relationships that could be construed as a potential conflict of interest.

## Publisher's Note

All claims expressed in this article are solely those of the authors and do not necessarily represent those of their affiliated organizations, or those of the publisher, the editors and the reviewers. Any product that may be evaluated in this article, or claim that may be made by its manufacturer, is not guaranteed or endorsed by the publisher.
