# Supplementary material for: Examining Effectiveness and Predictors of Treatment Response of Pivotal Response Treatment in Autism: An Umbrella Review and a Meta-Analysis
Source: Front Psychiatry. 2022 Jan 27;12:766150. doi: 10.3389/fpsyt.2021.766150 (PMC8830537; doi:10.3389/fpsyt.2021.766150)
Supplement: Supplementary file 1 [file Table_1.DOCX]

**Supplementary Material**

Supplementary Table 1. PsycINFO Search Strategy

|  | **Autism Spectrum Disorder Terms** |
| --- | --- |
| 1 | (autism or autistic or asperger* or rett syndrom* or retts syndrom* or rett disease or retts disease or morbus rett or kanner syndrom* or pervasive development* disorder* or pdd or pddnos or childhood disintegrative disorder* or cdd or hellers syndrom* or heller syndrom* or disintegrative psychosis).mp. |
| 2 | exp autism spectrum disorders/ |
| 3 | 1 or 2 |
|  | **Pivotal Response Treatment Terms** |
| 4 | (pivotal or prt).mp. |
| 5 | (naturalistic adj3 (teach* or paradigm* or intervention* or treatment* or communication or approach or development or therap* or training)).mp. |
| 6 | ((natural language or responsive) adj2 (teach* or paradigm* or intervention* or treatment* or communication or approach* or development or therap* or training)).mp. |
| 7 | ndbi.mp. |
| 8 | 5 or 6 or 7 |
| 9 | (self adj1 (initiat* or manag*)).mp. |
| 10 | (self directed adj2 (learning or intervention*)).mp. |
| 11 | ((child or children) adj3 initiat*).mp. |
| 12 | (responsiv* adj2 (multiple cue or multiple cues)).mp. |
| 13 | 9 or 10 or 11 or 12 |
| 14 | ((language* or communicat* or social) adj1 (functional or acquisition or acquir* or initiat*)).mp. |
| 15 | (language adj2 (intervention* or treatment* or therap* or training)).mp. |
| 16 | (social skill* adj3 train*).mp. |
| 17 | ((behavior* or behaviour*) adj (skill or skills) adj3 train*).mp. |
| 18 | 14 or 15 or 16 or 17 |
| 19 | ((parent or parents or parental) adj (train* or intervention*)).mp. |
| 20 | (parent* implement* adj3 early intervention*).mp. |
| 21 | 19 or 20 |
| 22 | 3 and (4 or 8 or 13 or 18 or 21) |
|  | **Randomized Controlled Trial Terms** |
| 23 | (random* or clinical trial* or controlled trial* or placebo or blind* or doubleblind or quasirandom* or control group*).mp. |
| 24 | 22 and 23 |
| 25 | limit 24 to english language |
| 26 | limit 25 to all journals |

Supplementary Table 2. Study level quality indicators and other study characteristics

| **Study** | **Country** | **Inclusion/Exclusion Criteria** | **Enrollment to Completion** | **Parent and clinician characteristics** |  | **Fidelity** | **Randomization** | **Blinding** | **Attrition** |
| --- | --- | --- | --- | --- | --- | --- | --- | --- | --- |
|  |  |  |  | **PRT** | **Other** |  |  |  |  |
| Barrett et al. (2020) | USA | Child: (i) 1.5-4.5 years of age; (ii) > ADOS-2 cut-off; (iii) meeting DSM-5 criteria based on expert clinical judgment Parent: willing to (i) complete 2-day intake and post-assessment battery; (ii) take part in 2h/pw sessions; (iii) available for 8hr/pw of clinician-delivered intervention. | Initial Assessment: 31; Randomized: 28; Allocated to Treatment: 16; Completed FU: 12 Allocated to Other: 12; Complete FU: 9. | NR | NR | Mean= 85.13% (SD= 12.07%); 83.33 % of families had PRT fidelity threshold ≥ 80 %. | Yes | Not complete/not presented | No |
| Gengoux et al. (2019) | USA | Inclusionary: (i) 2-5 years of age; (ii) ADI-R, ADOS-2, DSM-5 and expert clinical judgment; (iii) delayed on PLS-5 ≥ 1, 2 and 3SD below the mean for 2 & 3, 4 and 5 year-old children; (iv) at least one parent available to participate; Exclusionary: (i) > 1hr/pw individual speech therapy, > 14hr/pw 1:1 ABA; (ii) unstable treatment 1 month prior baseline; (iii) anticipated treatment changes during treatment; (iv) severe psychiatric or genetic syndrome; (v) primary language not English. | Eligibility Screening: 144; Enrolled: 93; Randomized: 48; Allocated to PRT: 24; Completed PRT:  FU: 23; Allocated to Other: 24; Complete FU: 20. | NR | NR | 91% of parents met the fidelity at week 24 (no parent met at baseline) | Yes | Yes | No |
| Hardan et al. (2015) | USA | Inclusionary: (i) 2-6 years of age; (ii) ADI-R, ADOS-2 and expert clinical judgment; (iii) delayed on PLS-5 ≥ 1, 2 and 3SD below the mean for 2 & 3, 4 and 5 year-old children; (iv) able to vocalize with intent; (v) stable treatment 1 month prior to baseline; (vi) at least one parent available to participate; Exclusionary: (i) > 1hr/pw individual speech therapy, > 14hr/pw 1:1 ABA; (ii) unstable treatment 1 month prior baseline; (iii) anticipated treatment changes during treatment; (iv) severe psychiatric or genetic syndrome; (v) primary language not English. | Eligibility Screening: 135; Enrolled: 104; Randomized: 53; Allocated to PRT: 27; Completed PRT FU: 25; Allocated to Other: 26 Complete FU: 22. | NR | NR | 84% of parents met the fidelity at week 12 (no parents met at baseline). | Yes | Yes | No |
| McDaniel et al. (2020) | USA | As Gengoux et al. (2019). | As Gengoux et al. (2019), | NR | NR | NR | Yes | Yes | No |
| Mohammadzaheri et al. (2014) | Iran | Inclusionary: (i) DSM-IV TR diagnosis confirmed by the clinician prior to the start of the study; (ii) placed in special education classroom; (iii) MLU ≥ 2 words; (iv) no hearing or vision impairments; (v) no psychiatric disorders; (vi) not bilingual; (vii) IQ ≥ 50. | Randomized to PRT: 15; Randomized to Other: 15. | NR | NR | Mean= 85% (range: 80-90%) | Not complete/not presented | Yes | No |
| Mohammadzaheri et al. (2015) |  | Inclusionary: (i) DSM-IV TR diagnosis confirmed by clinician prior to the start of the study; (ii) placed in special education classroom; (iii) MLU ≥ 2 words; (iv) no hearing or vision impairments; (v) no psychiatric disorders; (vi) not bilingual; (vii) IQ ≥ 50. | Randomized to PRT: 15; Randomized to Other: 15. | NR | NR | NR | Not complete/not presented | Yes | No |
| Nefdt et al. (2010) | USA | Child: (i) < 60 months of age; (ii) DSM-IV TR ASD diagnosis; (iii) < 20 functional words; Parent: (i) no prior PRT training; (ii) waiting for services; (iii) access to video recorded and DVD player | Enrolled: 34; Completed the study: 27. | NR | NR | Mean= 75.35% (SD= 26.61) | Not complete/not presented | Yes | Not complete/not reported |
| Schreibman & Stahmer (2014) | USA | Child: (i) ADI-R and ADOS confirmed ASD diagnosis; (ii) < 48 months old; (iii) no ID, neurological impairments nor major sensory impairments; (iv) no prior PRT not PECS treatment Parent: (i) willingness to participate in training and refrain from non-assigned treatments. | Randomized: 41;  Not completed: PRT: 1;  Not completed Other 1. | NR | NR | > 80% | Yes | Yes | No |
| Vernon et al. (2019) | USA | Child: (i) 1.5-4.5 years of age; (ii) > ADOS-2 cut-off; (iii) meeting DSM-5 criteria based on expert clinical judgment Parent: willing to (i) complete 2-day intake and post-assessment battery; (ii) take part in 2h/pw sessions; (iii) available for 8hr/pw of clinician-delivered intervention; (iv) participation in any existing interventions not an exclusionary criteria | Initial Assessment: 31; Randomized: 28; Allocated to Treatment: 16; Completed FU: 12; Allocated to Other: 12 Complete FU: 11. | NR | NR | Mean= 85.13% (SD= 12.07%); 83.33 % of families had PRT fidelity threshold ≥ 80 %. | Yes | Not complete/not presented | No |

Supplementary Table 3. Measurement proximity and measurement context quality indications

|  | Measure | Distal | Generalized | Parent Report | CME |
| --- | --- | --- | --- | --- | --- |
| Barrett et al. (2020) | SLO | Yes | Yes | No | No |
| Gengoux et al. (2019) | CDI | Yes | Yes | Yes | No |
|  | MSEL Expressive | Yes | Yes | No | No |
|  | MSEL Receptive | Yes | Yes | No | No |
|  | MSEL Composite | Yes | Yes | No | No |
|  | PLS-5 | Yes | YEs | No | No |
|  | SLO | Yes | Yes | No | No |
|  | VABS Daily Living Skills | Yes | Yes | Yes | No |
|  | VABS Expressive | Yes | Yes | Yes | No |
|  | VABS Receptive | Yes | Yes | Yes | No |
|  | VABS Socialization | Yes | Yes | Yes | No |
| Hardan et al. (2015) | CDI | Yes | Yes | Yes | No |
|  | PLS-5 | Yes | Yes | No | No |
|  | SLO | Yes | Yes | No | No |
|  | VABS Expressive | Yes | Yes | Yes | No |
|  | VABS Receptive | Yes | Yes | Yes | No |
| Mohammadzaheri et al. (2014) | SLO | Yes | Yes | No | No |
| Schreibman & Stahmer (2014) | CDI | Yes | Yes | Yes | No |
|  | MSEL Expressive | Yes | Yes | No | No |
|  | MSEL Receptive | Yes | Yes | No | No |
|  | MSEL Composite | Yes | Yes | No | No |
| Vernon et al. (2019) | MSEL Expressive | Yes | Yes | No | No |
|  | MSEL Receptive | Yes | Yes | No | No |
|  | MSEL Composite | Yes | Yes | No | No |
|  | PLS-5 | Yes | Yes | No | No |
|  | VABS Daily Living Skills | Yes | Yes | Yes | No |
|  | VABS Socialization | Yes | Yes | Yes | No |

Note: CDI: MacArthur-Bates Communicative Development Inventories; CME: correlated measurement error;

MSEL: Mullen Scales of Early Learning; PLS-5: Preschool Language Scale, Fifth Edition; SLO: Structured Language

Observation; VABS: Vineland Adaptive Behavior Scales
